# Comparison of Oral Zinc Sulfate with Systemic Meglumine Antimoniate in the Treatment of Cutaneous Leishmaniasis

**DOI:** 10.1155/2011/269515

**Published:** 2011-07-06

**Authors:** Mohamad Javad Yazdanpanah, Mahnaz Banihashemi, Fakhrozaman Pezeshkpoor, Mohammad Khajedaluee, Sororozaman Famili, Iman Tavakoli Rodi, Hadis Yousefzadeh

**Affiliations:** ^1^Research Center for Skin Disease and Cutaneous Leishmaniasis, Ghaem Hospital, School of Medicine, Mashhad University of Medical Sciences (MUMS), Mashhad, Iran; ^2^Department of Social Medicine, Mashhad University of Medical Sciences (MUMS), Mashhad, Iran; ^3^Mashhad University of Medical Sciences (MUMS), Mashhad, Iran; ^4^Young Researcher's Club, Islamic Azad university, Mashhad branch, Iran

## Abstract

The purpose of this study was to investigate comparison between oral zinc sulfate and meglumine antimoniate in the treatment of cutaneous leishmaniasis (CL). So 100 patients with CL were included and randomly divided into two groups. The first group was treated with oral zinc sulfate (10 mg/kg/day during 45 days period), and the second group was treated with systemic meglumine antimoniate (20 mg/kg/day intramuscularly for 20 days). Acceptable cure after completing 45 days of followup occurred in 30.2% of lesions in first group, while this was 35.5% for the second group. There is not any significant difference between the two treatment groups (*P* = 0.42). Serious side effects resulting in treatment discounting occurred in only meglumine antimoniate group. Although cure rate of systemic meglumine antimoniate group was better the treatment with zinc sulfate is much easier, cheaper, more convenient in consumption, safer, and nearly close cure percentage to systemic meglumine antimoniate injections without serious side effect.

## 1. Introduction

Cutaneous leishmaniasis (CL) is an important health problem in endemic area [1]. The incidence of leishmaniasis is in excess of 400000 cases annually, and 35 millions in 88 countries are at risk [2, 3]. In Iran, Mashhad City of Khorasan Razavi state is an endemic location for CL, and the most common form of CL (91% cases) in Mashhad is the dry form that the caused by Leishmania tropica [4]. Although CL is a self healing disease, it can result in disfiguring scar and long-lasting stigmas, which may destroy underlying structures like the nose, ear, or exposed sites of skin that cause psychological suffering of patients [3]. Several therapies proposed for CL [1], but between these treatments pentavalent antimonials, sodium stibogluconate (pentostam), and meglumine antimoniate (Glucantime; MA) have maximum efficacy in CL treatment, but serious side effects, high costs, multiple injections, and incomplete efficacy make researchers find replaceable therapies [5, 6]. Recently zinc sulfate (ZS) activities against *L. major* and *L. tropica* amastigotes were tested in vitro [7]. These results for CL were confirmed in animal models [8, 9]. Also recently ZS intralesional injections [10, 11] and oral ZS treatment [12] showed its effectiveness in CL treatment. With respect to harmlessness, minimum serious side effect, low cost, and easy consuming way of ZS this study was designed to evaluate comparison of oral ZS and MA in old world cutaneous leishmaniasis treatment. 

## 2. Patients and Methods

This prospective interventional case control clinical trial was performed in the Dermatology Department, Ghaem Hospital, Mashhad University of Medical Sciences, between October 2006 and May 2008. Patients with proven acute CL by a positive direct smear were selected for this study. A careful history was taken by the same dermatologist, and lesions were examined. Inclusion criteria included: duration of lesions less than 6 months and no antileishmaniasis treatment received during 2 month ago. Cases of pregnant or nursing women, patients with hepatic, renal, and heart diseases were excluded. This protocol was approved by the Ethical Committee of Mashhad University of Medical Sciences (MUMS). The planned treatment was explained to each patient, and consent was taken. 

### 2.1. Treatment and Followup

Using simple randomization patients were assigned into two treatment groups. First group received oral zinc sulfate (ZS) in a dose of 10 mg/kg/day during 45-day period before meal in three divided times, and second group received systemic Meglumine antimoniate (Glucantime; MA, Specia, Paris, France) 20 mg/kg/day intramuscularly for 20 days with a maximum of 3 vials of Glucantime. Patients were followed up during and at the end of clinical treatment. Also it was carried out after 45 days from completing treatment period. At each visit all lesions were reexamined by the same dermatologist. The size and indurations' of lesions were measured by palpation and ruler. In aspect of response to treatment in comparison with first visit patients graded as 4 improvements rate groups: 1: slight, 2: mild, 3: moderate, and 4: total clearance that in four respected groups decreased indurations of lesion were up to 25%, 25–50%, more than 50%, and less than 75% and 75% or more improvement or complete reepithelialization without any indurations considered as acceptable cure. 

### 2.2. Statistical Analysis

Statistical analyses were performed using SPSS Software (Version 11.5). Data were expressed as mean ± SD. Between groups, comparisons were made using independent *t*-test and Mann-Whitney test (for numerical variables) or Chi-square (*χ* ^2^) and Fisher exact test (for ordinal variables). A two-tailed *P* value of *P* < 0.05 was considered statistically significant for all calculations. 

## 3. Results

A total of 115 patients with proven CL participated in this study. From them 100 patients completed the treatment with followup visits. Six patients who received systemic MA were excluded because of side effects that include severe muscular pain and topical reaction of inoculation site (severe erythema and pruritus). In the present study five patients in MA group and 4 in ZS group were not followed up for the full treatment period so these 9 cases also dropped out. Finally 26 patients with 43 lesions were treated with ZS and 74 patients with 127 lesions with MA.

The characteristics of CL studied patients are presented in Table 1. There is no significant correlation between improvement rate and age of patients (*P* = 0.16) and duration of lesion (*P* = 0.09). Incomplete treatment (6 cases) because of side effects was seen just in MA group, and ZS had not any significant side effects (*P* < 0.05). Number of CL lesions localization in studied patients between two different treatments is shown in Table 2. Also there is normal distribution between CL involved sites (trunk, head and neck, upper limb, and lower limb) into two different treatment groups (ZS and MA) (*P* < 0.05).

**Figure 1:**
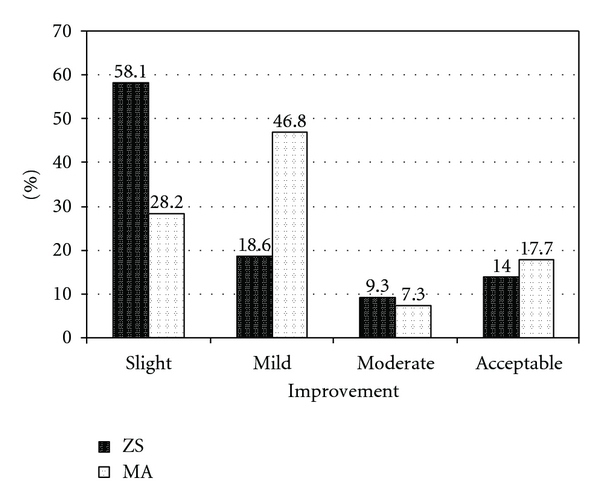
Response into two different treatments (ZS and MA) in CL studied patients after completing treatment. There is not any statistically significant difference between MA and ZS treatment groups in acceptable cure (*P* = 0.44).

**Figure 2:**
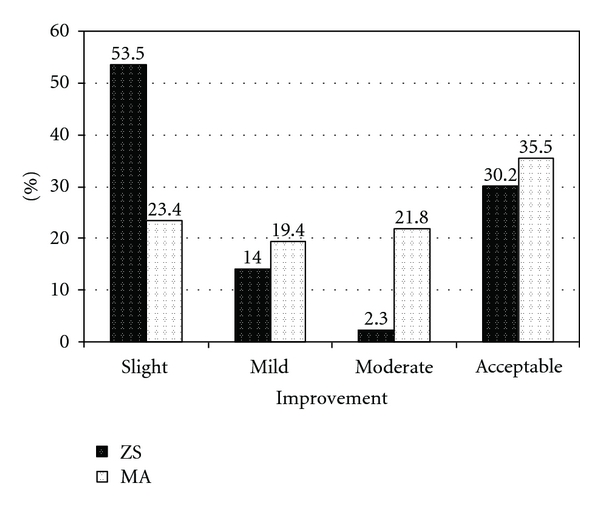
Response into two different treatments (ZS and MA) in CL studied patients after 45 days of followup. There is not any statistically significant difference between MA and ZS treatment groups in acceptable cure (*P* = 0.42).

Figures 1 and 2 show responding into ZS and MA treatments in CL studied patients after completing treatment and after 45 days of followup, respectively. Improvement rate as previously described graded into 4 groups (slight, mild, moderate, and acceptable cure). There is not any statistically significant difference between two treatments for acceptable cure after completing and 45 days of followup of treatment (*P* = 0.44 and *P* = 0.42, resp.). 

## 4. Discussion

Oral zinc sulfate had been used for several skin disorders for many years with few side effects. Recently publications showed that promastigotes and amastigotes of *L. major* and *L. tropica* were sensitive to ZS in vitro and in animals [7]. Zinc level is important for T cell, neutrophil, and natural killer cell function. The antileishmanial effect of zinc may result from the inhibition of enzymes that are necessary for the parasites, and these effects are dose dependent [13]. Our study results showed that both treatments (ZS and MA) have moderate cure rate in CL. Acceptable cure at the end of completing and after 45 days of followup of treatment for MA was 17% and 35%, respectively, while it was 14% and 30.2% for ZS treatment, respectively. 

In Iraq, Sharquie et al. reported that the efficacy of intralesionally and oral ZS was 94.8% and 96.9%, respectively [10, 12]. Iraji et al. in comparative study reported that cure rate of intralesionally ZS solution was 83.8% and 60% for MA. Cure rate of oral ZS according to first CL treatment in Mashhad (our studied area) was 9% [14] and 34.5% [15] and 37.9% [16] for systemic MA. 

Efficacy of ZS in Yazdanpanah et al.' [14] study as the first pilot study in Mashhad on 22 cases was 9% without control group that was in contrast with previously reported study by Sharquie et al. [12], so, for better decisions about ZS effectiveness, we decided to evaluate ZS efficacy and comparison with MA. 

In our study the efficacy of MA was more than that of ZS after completing and 45 days of followup, but there is not any significant difference between two treatments as MA and ZS treatments had nearly equal efficacy for acceptable cure after completing and 45 days of followup after treatment. This is comparable to previous studies in Mashhad [15, 16], while Iraji et al. reported that efficacy of intralesional ZS was more than MA [11]. These differences in treatment improvement results may be caused by the difference between way of treatment method and also complete treatment as considered total clearance is different, as our results (35% for MA and 17% for ZS treatment) are so close to previous studies in Mashhad (~36.2% for MA and 9% for ZS) [15, 17]. Second probability factors for these seen differences may be due to parasite strains. Most of CL in Mashhad was caused by *L. tropica* [17], while in other reported studies on ZS treatment the most lesions were caused by *L. major* [10–12]. Finally anti CL therapeutic resistance (especially MA) was discussed-problem [18] so there is probability of ZS resistance in our studied area, but this needs to be confirmed by greater number of CL patient's population. In spite of drug differences, dietary patients regimes especially phytate enriched diets (that interfere with zinc absorption) affect oral ZS intake. Iranians have diets with high phytates, so this may be one effective factor to this improvement response in comparison with other studies [19]. 

This present study indicates that systemic MA injections in CL treatment were better than ZS but oral ZS is cheaper, more convenient is consumption, and nearly close cure percentage to systemic MA injections without serious side effects. Because there are no cited studies about therapeutic effect of oral ZS on CL caused by *L. tropica*, we supposed that ZS therapeutic effects should be confirmed by greater sample volume. 

## Figures and Tables

**Table 1:** General characteristics of CL studied patients between two treatment groups (MA and ZS).

Drug	Patients	Age, year (mean ± SD)	Males	Females	No. of lesions	Duration of lesions (month)
MA	74	25 ± 19	39	35	127	4 ± 1.5
ZS	26	32 ± 23	11	15	43	4.5 ± 1.5

**Table 2:** Number of CL lesions in different sites between two treatment groups.

Lesion site	ZS (10 mg/kg/day)	MA (20 mg/kg/day)
Head and neck	19 (44.3%)	56 (44.1%)
Upper limb	18 (44.9%)	56 (44.1%)
Lower limb	5 (11.6%)	14 (11%)
Trunk	1 (2.3%)	1 (0.8%)

Frequency percentages in CL studied patients (%).
